# The effect of continuous positive airway pressure (CPAP) application on airway problems in pediatric patients with subglottic stenosis who undergo balloon dilatation

**DOI:** 10.1186/s13741-024-00478-5

**Published:** 2025-01-23

**Authors:** Zeliha Tuncel, Şenay Göksu, Özlem Deligöz, Kemal Tolga Saracoglu, Abdulatif Albasha, Bushra M. Abdallah, Ayten Saracoglu

**Affiliations:** 1 Department of Anesthesia and Reanimation, Ümraniye Training and Research Hospital, University of Health Sciences, Istanbul, Turkey; 2 Department of Anesthesia and Reanimation, Haydarpaşa Numune Training and Research Hospital, University of Health Sciences, Istanbul, Turkey; 3 Department of Anesthesiology, ICU, and Perioperative Medicine, Hazm Mebaireek General Hospital, Hamad Medical Corporation, Doha, Qatar; 4 College of Medicine, QU Health, Qatar University, Doha, Qatar; 5 Department of Anesthesiology, ICU, and Perioperative Medicine, Hamad General Hospital, Hamad Medical Corporation, Doha, Qatar; 6 Department of Anesthesiology, ICU, and Perioperative Medicine, Aisha Bint Hamad Al-Attiyah Hospital, Hamad Medical Corporation, Doha, Qatar

**Keywords:** Pediatric airway, Subglottic stenosis, CPAP, Apneic ventilation

## Abstract

**Background:**

Subglottic stenosis is a significant clinical challenge in pediatric anesthesia, often necessitating interventions that can lead to various postoperative complications. The aim of this study was to determine the effect of prophylactic continuous positive airway pressure (CPAP) application on recovery time and airway complications in pediatric patients with subglottic stenosis undergoing balloon dilatation.

**Methods:**

A prospective, double-blinded, parallel-group, randomized controlled study was conducted at Health Sciences University Ümraniye Training and Research Hospital on pediatric patients with subglottic stenosis, aged from 0 to 12 years and who underwent elective balloon dilatation under general anesthesia. Patients were randomized in a 1:1 ratio into the CPAP or non-CPAP group. The primary outcome was the duration of recovery time. Secondary outcomes included bronchospasm, the number of desaturation episodes, intubation, tracheostomy, and the need for intensive care.

**Results:**

A total of 84 patients were enrolled in this randomized controlled trial, 81 of which received the allocated treatment and were analyzed (non-CPAP *n* = 41, CPAP *n* = 40). Compared to controls, the proportions of bronchospasm, tracheal secretion, need for intensive care, and tracheostomy were consistently lower in the CPAP group, whereas the requirement of intubation was higher. Further, the mean recovery time was significantly shorter in the CPAP group compared to the non-CPAP group (mean difference − 3.3 min, 95%CI − 5.16 to − 1.44, *p* = 0.0007). Despite lacking statistical significance, the CPAP group had reduced odds of developing bronchospasm, tracheal secretion, need for intensive care, and tracheostomy, but higher odds of requiring intubation when compared to the controls.

**Conclusion:**

Prophylactic CPAP application following therapeutic balloon dilatation in pediatric patients who have developed subglottic stenosis due to acquired or congenital causes appears to effectively shorten recovery time and may have a role in decreasing postoperative pulmonary complications; however, more research is recommended to further confirm these findings.

**Trial registration:**

The protocol for this clinical trial was retrospectively registered on clinicaltrials.gov with registration ID NCT06183515 on 30 November 2023.

**Supplementary Information:**

The online version contains supplementary material available at 10.1186/s13741-024-00478-5.

## Introduction

Acquired pediatric subglottic stenosis is reported to have a prevalence of 1–2% (Cantarella et al. 2020). This rate reaches 8.3% in different case series (Percul et al. 2023). Acquired pediatric subglottic stenosis is commonly attributed to prolonged intubation, whereas the etiology also involves a history of traumatic or difficult airway (Powell et al. 2020). Balloon dilatation laryngoplasty is an efficient and safe technique for treating primary and secondary acquired laryngotracheal stenosis (Hautefort et al. 2012). It is successfully applied in these children. Airway surgery typically necessitates a deeper level of anesthesia to control airway reflexes and manage the fluctuations in hemodynamic parameters, which are characteristic of this surgery.

Nevertheless, general anesthesia must be applied several times to patients due to the need for multiple balloon dilatations. At the same time, the procedure also necessitates coping with postoperative complications that may arise. Because alveolar collapse, which is related to general anesthesia, impairs gas exchange by creating a shunt effect, it potentially increases perioperative hypoxemic episodes (Habre et al. 2017; Bonasso et al. 2019), which in turn increases the risk for postoperative pulmonary complications. A recent retrospective analysis found that 40.6% of children who underwent balloon dilatation experienced desaturation. Additionally, tracheotomy was required in 15.6% of cases, with an equal percentage needing tracheal intubation (Tuzuner et al. 2022).

Nasal continuous positive airway pressure (CPAP) acts as a “pressure” bridge between spontaneous breathing and controlled mechanical ventilation. As a result, there is an increasing trend in the prophylactic use of nasal CPAP in pediatric patients following high-risk airway procedures to reduce postoperative airway complications. The procedure has been shown to improve oxygenation by reducing the alveolar-arterial oxygen difference after pediatric laparoscopic surgery (Abdel-Ghaffar et al. 2019). However, it has been proven that CPAP applied after pediatric cardiac surgery has favorable effects on peak expiratory flow (Silva et al. 2016). Still, there is no study published on the prophylactic use of CPAP after balloon dilatation in children with tracheal stenosis. Therefore, the objective of this study was to compare recovery time, the requirement for intensive care, tracheostomy, intubation, the number of desaturation episodes, and the rate of airway complications including bronchospasm and tracheal secretions, in pediatric patients with subglottic stenosis who received CPAP versus those who did not receive CPAP.

### Methods

#### Study design and participant selection

This prospective, double-blinded, parallel-group, randomized controlled clinical study was conducted between the 1st of January 2022 and the 1st of October 2022 at Health Sciences University Ümraniye Training and Research Hospital in Turkey. The study protocol received ethical approval from the Umraniye Training and Research Hospital Ethics Committee with decision number 35 dated 16th of December 2021 (approval number: B.10.1.TKH.4.34.H.GP.0.01/352). Before the conduct of the study or any study-related procedures, written informed consent was obtained from all legal guardians of the participants. The study was designed following the Declaration of Helsinki defined in 2013 (World Medical Association 2013) and the CONSORT 2010 statement (Schulz et al. 2010; Moher et al. 2010).

The study included pediatric patients who were 0 to 12 years of age, classified as II and III according to the American Society of Anesthesiologists (ASA) physical status classification, and who underwent elective subglottic balloon dilatation under general anesthesia due to acquired or congenital subglottic stenosis.

Patients with congenital or acquired diseases of the primary lung or choanal atresia, those younger than 38 gestational weeks, those older than 12 years, and intubated patients were excluded from the study.

#### Randomization, allocation concealment, and blinding

Patients were randomized in a 1:1 ratio by the principal investigator through computer-generated simple randomization into two groups: the control non-CPAP group (*n* = 42) and CPAP group (*n* = 42). Randomization was performed with the Random Integer Generator software (https://www.random.org/). Group allocations were concealed securely in a password-protected computer and were only disclosed to an attending anesthesiologist who was not directly involved in the study. To eliminate potential bias, this was a double-blinded study, where the investigators, analysts, and participants were not aware of the group assignments.

#### Study procedures and interventions

Before the operation, the 6–4-3–1 regimen (6 h for solids, 4 h for formula and non-human milk, 3 h for breast milk, and 1 h for clear fluids) was followed for fasting (Frykholm et al. 2022). No patient received premedication. We conducted standard patient monitoring, which involved the use of electrocardiogram (ECG), heart rate (HR) assessment, end-tidal carbon dioxide (ETCO_2_) measurement, pulse oximetry (SpO_2_), and temperature monitoring. To achieve anesthesia induction, we administered 100% oxygen via an anesthesia mask to infants and children aged 0 to 2 years while ensuring the preservation of spontaneous breathing at the beginning of anesthesia. Additionally, Sevoflurane inhalation anesthesia was administered at a concentration of 2% to achieve a 1 minimum alveolar concentration (MAC). Patients aged 2 to 12 were intravenously given Propofol 1–2 mg/kg. After a thorough evaluation of vocal cord and respiratory tract anatomy and dynamic airway assessment by the otolaryngologist, intravenous Fentanyl was administered at a dose of 0.5 µg/kg to the patient while maintaining spontaneous breathing. In patients for whom continuous airway control was not desired, a dose of 0.6 mg/kg Rocuronium was administered as necessary.

On the other hand, during the maintenance phase, patients for whom the preservation of spontaneous respiration was desired, intravenous Propofol at a rate of 0.5 to 1 mg/kg/h and Remifentanil at a rate of 0.03 mg/kg/min were administered through titration. In all patients, during the balloon dilatation procedure, a transition to apneic respiration was initiated, and a nasal endotracheal tube (ETT) size 2 (trimmed to the appropriate length based on the patient’s age and weight) was used for oxygen insufflation. An SpO_2_ level below 94% was considered the controlled hypoxemia threshold, where point active ventilation with a mask was initiated, allowing the surgical team to continue with the procedure, and we could return to apneic ventilation. In patients whose hypoxia persisted during mask ventilation, endotracheal intubation was conducted to restore normoxia and normocapnia. Following this, apneic ventilation was resumed. During the procedure, all patients were administered intravenous Paracetamol at a dose of 10 mg/kg, Prednisolone at 1 mg/kg, and, as needed, Theophylline at 3 mg/kg and/or Magnesium Sulfate at 10 mg/kg.

In cases where desaturation occurred after the procedure, patients were either intubated with an appropriately sized uncuffed endotracheal tube based on their age and the level of tracheal stenosis or in patients with tracheostomy tubes, their secretions were aspirated. Intravenous Sugammadex was administered at 3 mg/kg to reverse the neuromuscular effects at extubation. At the end of the procedure, patients suitable for extubation or decannulation were monitored on spontaneous breathing.

During the postoperative period, the CPAP group received FiO_2_ of 60% and 8 to 12 mmHg of nasal CPAP, or CPAP was initiated through the tracheostomy cannula. The non-CPAP group received FiO_2_ of 60%, and oxygen support was provided at a rate of 3 L/min either via mask for those who were extubated or through a T-piece for those with a tracheostomy cannula. In the presence of desaturation, FiO_2_ was increased to 80% in both groups. Patients who experienced intercostal retractions, persistent dyspnea, and unresolved desaturation within 60 min of observation in the recovery unit were transferred to the pediatric intensive care unit. These patients were either re-intubated or provided invasive/non-invasive mechanical ventilation support through the tracheostomy cannula when needed.

#### Primary and secondary outcomes

The primary outcome was recovery time, measured in minutes. Secondary outcomes were (1) number of desaturation episodes with oxygen saturation < 90%, (2) development of bronchospasm, (3) development of tracheal secretions, (4) requirement for intensive care, (5) requirement for tracheostomy, and (6) requirement for re-intubation.

Recovery time in this study was defined as the period during which the patient was monitored under full observation in the operating theater (OT) following the procedure, as these patients cannot be directly sent to the post-anesthesia care unit (PACU) due to their condition. Hence, the recovery time was the time from Propofol discontinuation to extubation. Patients were transferred to PACU only after full recovery in the OT, and those requiring further critical care were transferred to the intensive care unit (ICU). Thus, recovery time is a vital measure of postoperative stabilization, and CPAP’s potential in reducing this time is of clinical importance.

#### Data collection and variables

The demographic data of all patients, including sex, age, weight, subglottic stenosis grade, and previous intubation history, were recorded. The grade of subglottic stenosis was determined according to the Myer and Cotton classification. Throughout the procedure, heart rate, mean arterial pressure and oxygen saturation (SpO_2_) were recorded at five different time intervals (T_0_: before the procedure, T_1_: 5th minute, T_2_: 10th minute, T_3_: 15th minute, and T_4:_ 20th minute of the procedure). Following the procedure, data on the study outcomes, including bronchospasm, intubation, tracheostomy, desaturation episodes, duration of recovery time, and the need for intensive care were documented.

#### Statistical analysis

The power analysis was conducted using the G*power 3.1 program for our study. We used an alpha error of 0.05, a power of 0.80, and an effect size of 0.67, which was calculated based on an expected mean difference showing a 10% reduction in recovery time, which—for an average recovery time of around 30 min, as has been noted for this patient population in our hospital—is equivalent to a reduction by 3 min. The total required sample size was determined to be 72 (72/2 groups = 36 patients per group). However, to account for the possibility of ineligible participants requiring exclusion, the number of recruited participants per group was increased by 15%. Hence, the total number of enrolled participants per group was 42.

In the evaluation of the data, descriptive statistical methods were employed, including mean, standard deviation, median, and interquartile range. The distribution of variables was assessed using the Shapiro–Wilk test. The primary outcome was analyzed using independent samples *t* test while the secondary outcomes were analyzed using logistic regression models. We reported the mean difference, odds ratios (OR), 95% confidence intervals (95%CIs), and the exact *p*-values. All logistic regression models were tested for link specification using the *linktest* and model fit using McFadden’s test. All statistical analyses in this study were conducted according to the per-protocol principle using the NCSS (Number Cruncher Statistical System) 2007 Statistical Software package (UT, USA) and Stata 18.0 (StataCorp, College Station, TX, USA).

## Results

**Fig. 1:**
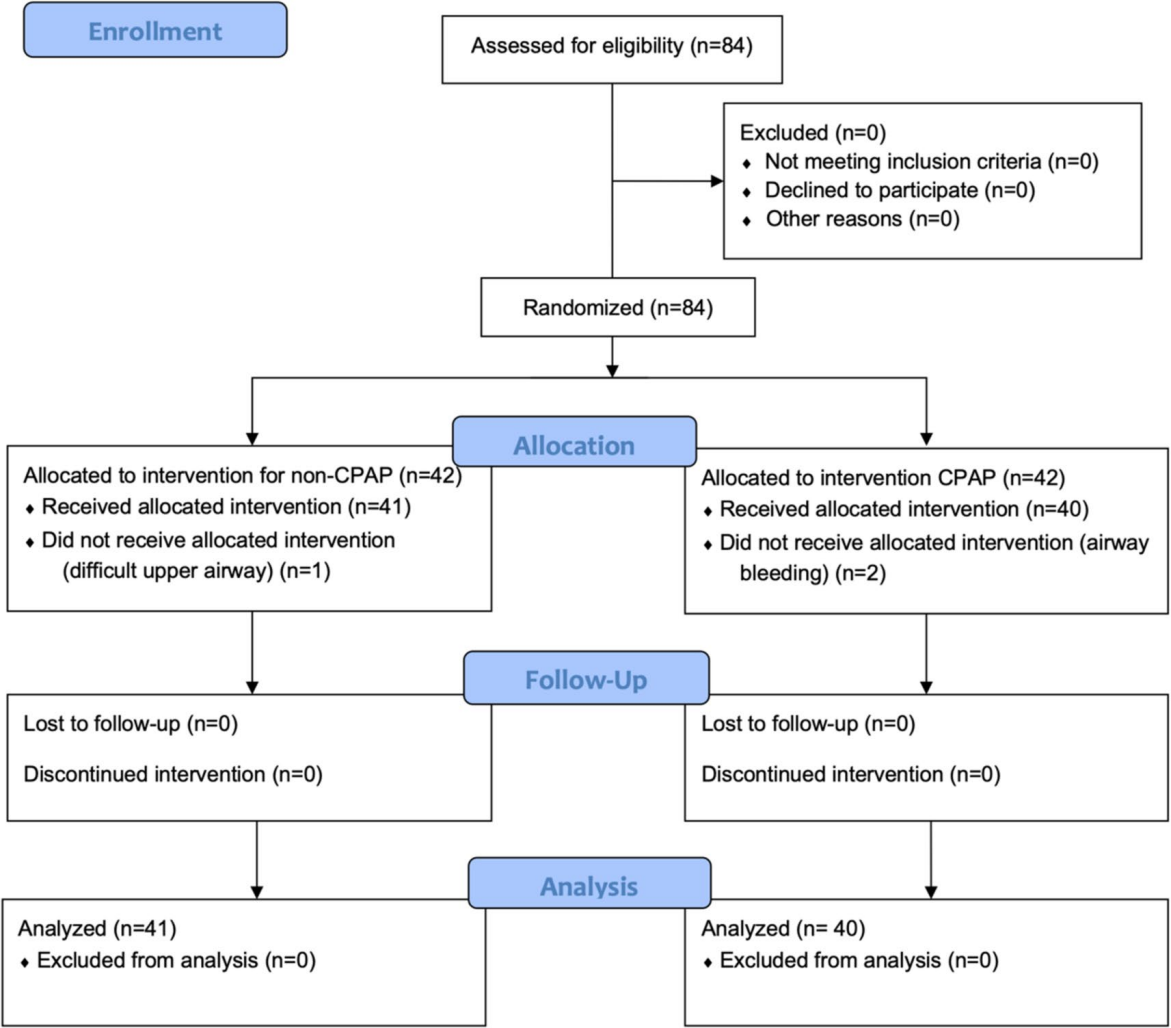
CONSORT flow diagram for patient recruitment and follow-up

A total of 84 patients were enrolled in the study and underwent randomization. Following allocation, two patients in the CPAP group did not receive the allocated intervention and were excluded due to airway bleeding, and one patient in the non-CPAP group was excluded because of a cystic structure causing upper airway constriction. Data belonging to the remaining 81 participants who received the allocated intervention were analyzed (Fig. 1).

The demographic characteristics of the patients are shown in Table 1. The groups did not show any significant difference regarding the distribution of patients according to age, sex, duration of operation, and grade of subglottic stenosis. The mean age of the participants was 1.89 and 2.67 years in the non-CPAP and CPAP groups, respectively. Both groups had more male participants than females. The mean duration of surgery was almost equal in both groups. The CPAP group was mostly comprised of participants with grades 2 and 3 subglottic stenosis (48% each), whereas the control group had more participants with grade 3 subglottic stenosis (65%).

**Table 1:** Demographic characteristics of the participants

Variable	Level	Non-CPAP (*n* = 41)	CPAP (*n* = 40)	*p* value
Age, years		1.89 ± 2.08	2.67 ± 3.19	0.88
Age, years		1 (0.37–3)	1 (0.27–4)	
Sex	Female	16 (39%)	18 (45%)	0.59
	Male	25 (61%)	22 (55%)	
Weight, kg		9.2 ± 4.4	10.7 ± 7.6	0.30
Duration of surgery, min		28.8 ± 3.8	28.6 ± 4.0	0.79
Subglottic stenosis grade	Grade 2	14 (35%)	19 (48%)	0.15
	Grade 3	26 (65%)	19 (48%)	
	Grade 4	0 (0%)	2 (5%)	

Data are presented as number (percentage), mean ± standard deviation, or median (Q1–Q3). *CPAP* continuous positive airway pressure, *SD* standard deviation, *IQR* interquartile range

Table 2 outlines the different outcomes studied in the CPAP and non-CPAP groups. The mean recovery time was significantly shorter in the CPAP group compared to the non-CPAP group (mean difference − 3.3 min, 95%CI − 5.16 to − 1.44, *p* = 0.0007). No difference was observed in the number of desaturation episodes between the two groups. Additionally, the incidence of complications such as bronchospasm, tracheal secretion, need for intensive care, and tracheostomy was consistently lower in the CPAP group. However, the proportion of patients requiring intubation was higher in the CPAP group compared to the non-CPAP group (25% vs 17%, respectively).

**Table 2:** Comparison of the outcomes between both groups

Variable	Non-CPAP (*n* = 41)	CPAP (*n* = 40)	Effect size (95%CI)	*p-*value
Primary outcome				
Recovery time, min	36.1 ± 4.4	32.8 ± 4.0	− 3.3^*a*^ (− 5.16 to − 1.44)	0.0007
Secondary outcomes				
Desaturation episodes	2.63 ± 0.7	2.58 ± 0.9	− 0.05^*a*^ (− 0.41–0.31)	0.781
Bronchospasm	10 (24%)	5 (12%)	0.44^b^ (0.14–1.44)	0.175
Tracheal secretion	12 (29%)	10 (25%)	0.81^b^ (0.30–2.15)	0.666
Need for intensive care	12 (29%)	10 (25%)	0.81^b^ (0.30–2.15)	0.666
Tracheostomy	7 (17%)	5 (12%)	0.69^b^ (0.20–2.40)	0.564
Intubation	7 (17%)	10 (25%)	1.62^b^ (0.55–4.78)	0.383

Data are presented as number (percentage), or mean ± standard deviation. *CPAP* continuous positive airway pressure, *95%CI* 95% confidence interval

^a^Mean difference

^b^Odds ratio

After performing logistic regression, it was found that the CPAP group had reduced odds of developing bronchospasm, tracheal secretion, need for intensive care, and tracheostomy compared to the control group (Table 2). However, the CPAP group had higher odds of requiring intubation (OR 1.62, 95%CI 0.55–4.78, *p* = 0.383). Despite these clinically important findings, there was a lack of statistical significance, possibly due to the small sample size.

Hemodynamic data of the participants at different timepoints during the procedure is presented in Supplementary Table S1. No significant difference was observed between the CPAP and control groups regarding the mean heart rate, mean arterial pressure, and SpO_2_ measured during the procedure. Supplementary Table S2 outlines the intragroup comparisons of the hemodynamic data. In intragroup comparisons, significant changes were observed in the mean heart rate values measured at the T_0_, T_1_, T_2_, T_3_, and T_4_ timepoints during the procedure in both groups. The mean heart rate values at time T_0_ were significantly lower than those measured at T_1_, T_2_, T_3_, and T_4_. Significant changes were observed in the mean SpO_2_ levels at T_0_, T_1_, T_2_, T_3_, and T_4_ during the procedure in both groups. The mean SpO_2_ level at T_0_ was significantly higher compared to the mean SpO_2_ at T_1_, T_2_, and T_3_ (Supplementary Tables S1–S2).

## Discussion

In this prospective, randomized, controlled study, we investigated the effects of postoperative prophylactic CPAP application on airway complications and recovery time in pediatric patients with subglottic stenosis who underwent balloon dilatation. While a significant reduction in recovery time was achieved with CPAP application, bronchospasm, suction requirements, intensive care admissions, and the need for tracheostomy were observed less frequently in these pediatric patients.

Anesthesia management in children with subglottic stenosis presents various challenges. These patients often have major comorbidities such as prematurity, lung parenchymal diseases, and central airway obstructions. Managing central airway obstruction and sharing the airway during surgical procedures can critically jeopardize airway management. Essential preoperative and postoperative preparations should be carefully planned in anticipation of the risk of encountering total airway obstruction.

Humphreys et al. (Humphreys et al. 2019) reported that in a cohort of 87 children who underwent dynamic airway evaluation, 34% experienced at least one hypoxemic event, and 23% of the procedures were interrupted for rescue oxygenation. In our study, the incidence of desaturation events during the procedure, defined as SpO_2_ dropping below 90%, was similar in both groups. The group receiving CPAP demonstrated a reduced incidence of postoperative pulmonary complications. Because the effects of pediatric CPAP application on hemodynamic tolerance, lung tissue, and postoperative atelectasis remain unclear, many pediatric anesthesiologists refuse to use it in their clinical practice (Acosta et al. 2021). However, although we observed hemodynamic changes during the surgical procedure in both groups in our study, it was noted that hemodynamic parameters remained stable during nasal CPAP applications.

Publications supporting the benefits of postoperative prophylactic nasal CPAP in pediatric patients are increasing day by day. In a recent systematic review and network meta-analysis, which included 1421 infants and young children, noninvasive respiratory support modes were compared to conventional oxygen therapy to prevent postoperative extubation failure (Iyer et al. 2023). As a result, it was demonstrated that CPAP reduces the incidence of extubation, and treatment failure more effectively compared to both high-flow nasal cannula (HFNC) and bilevel positive airway pressure (BiPAP). Another meta-analysis reported that CPAP was more effective than high-flow nasal cannula oxygenation in reducing the risk of treatment failure in infants aged 1 to 6 months (Luo et al. 2019). However, to the best of our knowledge, our study is the first investigation conducted in children who underwent balloon dilatation for subglottic tracheal stenosis.

According to UK Airway Intervention Registry data, approximately 22% of pediatric airway stenosis cases treated with balloon dilatation require tracheostomy (Powell et al. 2020). In our study, tracheostomy was performed in a total of 12 children, with 5 in the CPAP group and 7 in the control group (14.8%).

The primary outcome of our study is the reduction in recovery time observed in children who received postoperative CPAP. This outcome comes with several advantages. First, due to the larger body surface area in children, there is a higher tendency for heat loss. Therefore, prolonged recovery time has been associated with hypothermia and slow drug metabolism (Misal et al. 2016). Second, as the recovery period increases, the risk of emergence delirium increases. It has been reported that this phenomenon, which causes cognitive alterations, can develop with a frequency of 50% to 80% in pediatric anesthesia (Cascella et al. 2020). Third, the delayed oral hydration due to prolonged recovery time in these children can lead to agitation (Yin et al. 2020). The perioperative process should be safe, comfortable, adapted to special needs and non-traumatic, and all necessary precautions should be taken to minimize possible complications (Ciccozzi et al. 2022). Before dilatation, appropriate depth of anesthesia should be ensured to minimize respiratory effort because continued strong respiratory effort during balloon dilatation may cause airway injury, including possible negative pressure pulmonary edema (Gungor 2012). Considering all these indirect adverse effects, the necessity of prophylactic CPAP after pediatric airway interventions becomes better understood.

Recovery time was selected as the primary outcome because, while airway adverse events and respiratory complications are expected in these patients due to their pre-existing anatomical problems, recovery time reflects a broader measure of postoperative recovery and overall patient stability. Complications such as bronchospasm, suction requirements, or the need for tracheostomy were considered secondary outcomes, given their predictability in this population. However, recovery time allows us to assess whether CPAP can expedite the overall stabilization of these patients after surgery, which could, in turn, reflect fewer postoperative complications. A shorter recovery time implies faster stabilization and a reduced need for intensive postoperative care, which directly impacts patient outcomes and hospital resources.

Moreover, respiratory complications can influence recovery time, and prolonged recovery may increase the risk of developing respiratory adverse events. Therefore, recovery time serves as a more encompassing primary outcome since it may reflect both the direct effects of CPAP and the indirect influence of any complications.

Given that, in our study, the CPAP intervention was implemented for both sexes, across varying ages (0–12 years), and encompassing both acquired and congenital subglottic stenosis types, our findings indicate its potential benefits and promising implications for enhancing postoperative outcomes, which could be generalizable across a diverse pediatric population undergoing balloon dilatation.

The primary limitation of this study is the small sample size and the single-center design. While we calculated a powered sample size for the primary outcome, we did not perform a similar calculation for the secondary outcomes, which may have contributed to the lack of statistical significance observed in these findings. Larger, multicenter studies with adequately powered sample sizes for both primary and secondary outcomes are necessary to validate and expand upon these results. Secondly, due to the short surgical duration, changes in SaO_2_ in arterial blood gas could not be compared using invasive blood pressure monitoring. Lastly, carbon dioxide changes in the apneic and recovery periods could not be evaluated transcutaneously due to equipment insufficiency.

## Conclusion

Prophylactic CPAP application following therapeutic balloon dilatation in pediatric patients who have developed subglottic stenosis due to acquired or congenital causes appears to effectively shorten recovery time and decrease postoperative pulmonary complications.

## Supplementary Information

Supplementary Material 1. Supplementary Figure S1. CONSORT 2010 checklist of information to include when reporting a randomized trial. Supplementary Table S1. Hemodynamic data of the participants at different timepoints during the procedure. Supplementary Table S2. Intertemporal variation of hemodynamic data.

## Data Availability

The data used in this work are available upon reasonable request from the corresponding author.
