# Supplementary material for: The effect of continuous positive airway pressure (CPAP) application on airway problems in pediatric patients with subglottic stenosis who undergo balloon dilatation
Source: Perioper Med (Lond). 2025 Jan 23;14:10. doi: 10.1186/s13741-024-00478-5 (PMC11756109; doi:10.1186/s13741-024-00478-5)
Supplement: Supplementary file 1 — Supplementary Material 1. Supplementary Figure S1. CONSORT 2010 checklist of information to include when reporting a randomized trial. Supplementary Table S1. Hemodynamic data of the participants at different timepoints during the procedure. Supplementary Table S2. Intertemporal variation of hemodynamic data. [file 13741_2024_478_MOESM1_ESM.pdf]

Supplementary Material

The Effect of Continuous Positive Airway Pressure (CPAP) Application on Airway Problems in Pediatric Patients with Subglottic Stenosis Who Undergo Balloon Dilatation

Zeliha Tuncel[1], Şenay Göksu[1], Özlem Deligöz[2], Kemal Tolga Saracoglu[3,4], Abdulatif Albasha[5], Bushra M Abdallah[4], Ayten Saracoglu[4,6]

[1] University of Health Sciences, Ümraniye Training and Research Hospital, Department of Anesthesia and Reanimation, Istanbul, Turkey.

[2] University of Health Sciences, Haydarpaşa Numune Training and Research Hospital, Department of Anesthesia and Reanimation, Istanbul, Turkey.

[3] Department of Anesthesiology, ICU, and Perioperative Medicine, Hazm Mebaireek General Hospital, Hamad Medical Corporation, Doha, Qatar.

[4] College of Medicine, QU Health, Qatar University, Doha, Qatar.

[5] Department of Anesthesiology, ICU, and Perioperative Medicine, Hamad General Hospital, Hamad Medical Corporation, Doha, Qatar.

[6] Department of Anesthesiology, ICU, and Perioperative Medicine, Aisha Bint Hamad Al-Attiyah Hospital, Hamad Medical Corporation, Doha, Qatar.

Corresponding author:

Ayten Saracoglu,

Department of Anaesthesiology, ICU, and Perioperative Medicine, Aisha Bint Hamad Al-Attiyah Hospital, Hamad Medical Corporation, Doha, Qatar

P O BOX 3050

Doha, Qatar

Telephone number: +97433398131

Email: saracogluayten@gmail.com

CONSORT 2010 checklist of information to include when reporting a randomised trial*

Section/Topic	Item No	Checklist item	Reported on page No
**Title and abstract**			
	1a	Identification as a randomised trial in the title	1
	1b	Structured summary of trial design, methods, results, and conclusions (for specific guidance see CONSORT for abstracts)	2
**Introduction**			
Background and objectives	2a	Scientific background and explanation of rationale	3
	2b	Specific objectives or hypotheses	3
**Methods**			
Trial design	3a	Description of trial design (such as parallel, factorial) including allocation ratio	3 – 4
	3b	Important changes to methods after trial commencement (such as eligibility criteria), with reasons	NA
Participants	4a	Eligibility criteria for participants	3 – 4
	4b	Settings and locations where the data were collected	3 – 4
Interventions	5	The interventions for each group with sufficient details to allow replication, including how and when they were actually administered	4 – 6
Outcomes	6a	Completely defined pre-specified primary and secondary outcome measures, including how and when they were assessed	6
	6b	Any changes to trial outcomes after the trial commenced, with reasons	NA
Sample size	7a	How sample size was determined	6
	7b	When applicable, explanation of any interim analyses and stopping guidelines	NA
Randomisation:			
Sequence generation	8a	Method used to generate the random allocation sequence	4
	8b	Type of randomisation; details of any restriction (such as blocking and block size)	4
Allocation concealment mechanism	9	Mechanism used to implement the random allocation sequence (such as sequentially numbered containers), describing any steps taken to conceal the sequence until interventions were assigned	4
Implementation	10	Who generated the random allocation sequence, who enrolled participants, and who assigned participants to interventions	4
Blinding	11a	If done, who was blinded after assignment to interventions (for example, participants, care providers, those assessing outcomes) and how	4
	11b	If relevant, description of the similarity of interventions	4 – 6
Statistical methods	12a	Statistical methods used to compare groups for primary and secondary outcomes	6
	12b	Methods for additional analyses, such as subgroup analyses and adjusted analyses	6
**Results**			
Participant flow (a diagram is strongly recommended)	13a	For each group, the numbers of participants who were randomly assigned, received intended treatment, and were analysed for the primary outcome	6 – 7
	13b	For each group, losses and exclusions after randomisation, together with reasons	6 – 7
Recruitment	14a	Dates defining the periods of recruitment and follow-up	4
	14b	Why the trial ended or was stopped	NA
Baseline data	15	A table showing baseline demographic and clinical characteristics for each group	7 – 8
Numbers analysed	16	For each group, number of participants (denominator) included in each analysis and whether the analysis was by original assigned groups	8 – 9
Outcomes and estimation	17a	For each primary and secondary outcome, results for each group, and the estimated effect size and its precision (such as 95% confidence interval)	8 – 9
	17b	For binary outcomes, presentation of both absolute and relative effect sizes is recommended	8 – 9
Ancillary analyses	18	Results of any other analyses performed, including subgroup analyses and adjusted analyses, distinguishing pre-specified from exploratory	NA
Harms	19	All important harms or unintended effects in each group (for specific guidance see CONSORT for harms)	NA
**Discussion**			
Limitations	20	Trial limitations, addressing sources of potential bias, imprecision, and, if relevant, multiplicity of analyses	11
Generalisability	21	Generalisability (external validity, applicability) of the trial findings	11
Interpretation	22	Interpretation consistent with results, balancing benefits and harms, and considering other relevant evidence	9 – 11
**Other information**			
Registration	23	Registration number and name of trial registry	12
Protocol	24	Where the full trial protocol can be accessed, if available	12
Funding	25	Sources of funding and other support (such as supply of drugs), role of funders	12

*We strongly recommend reading this statement in conjunction with the CONSORT 2010 Explanation and Elaboration for important clarifications on all the items. If relevant, we also recommend reading CONSORT extensions for cluster randomised trials, non-inferiority and equivalence trials, non-pharmacological treatments, herbal interventions, and pragmatic trials. Additional extensions are forthcoming: for those and for up to date references relevant to this checklist, see www.consort-statement.org.

Supplementary Figure S1. CONSORT 2010 checklist of information to include when reporting a randomized trial.

Supplementary Table S1. Hemodynamic data of the participants at different timepoints during the procedure.

		Non-CPAP	CPAP	*p* Value
Heart Rate, BPM, mean (SD)	T_0	116.15 (11.87)	115.18 (14.32)	0.740
	T_1	123.83 (10.69)	123.25 (12.24)	0.821
	T_2	128.37 (10.81)	126.00 (11.77)	0.349
	T_3	126.24 (19.73)	126.85 (9.49)	0.861
	T_4	127.12 (7.86)	125.73 (6.73)	0.393
	p Value	0.0001	0.0001	
MAP, mmHg, mean (SD)	T_0	55.29 (5.14)	55.08 (6.15)	0.863
	T_1	53.56 (4.68)	53.03 (5.88)	0.651
	T_2	54.07 (4.09)	54.35 (5.16)	0.789
	T_3	55.22 (3.87)	54.55 (5.25)	0.515
	T_4	54.80 (3.99)	53.78 (5.05)	0.311
	p Value	0.0001	0.0001	
SpO_2, %, mean (SD)	T_0	94.24 (2.31)	94.78 (2.19)	0.292
	T_1	91.63 (2.40)	92.15 (1.92)	0.288
	T_2	90.59 (1.80)	90.75 (1.86)	0.687
	T_3	93.44 (1.64)	93.13 (1.60)	0.387
	T_4	96.71 (1.08)	96.48 (1.13)	0.347
	p Value	0.0001	0.0001	

CPAP, continuous positive airway pressure; MAP, Mean arterial pressure.

Supplementary Table S2. Intertemporal variation of hemodynamic data.

	Heart rate		MAP		SpO_2	
	Non-CPAP	CPAP	Non-CPAP	CPAP	Non-CPAP	CPAP
T_0 / T_1	0.0001	0.0001	0.0001	0.0001	0.0001	0.0001
T_0 / T_2	0.0001	0.0001	0.021	0.139	0.0001	0.0001
T_0 / T_3	0.001	0.0001	0.882	0.304	0.02	0.0001
T_0 / T_4	0.0001	0.0001	0.264	0.01	0.0001	0.0001
T_1 / T_2	0.001	0.009	0.280	0.016	0.051	0.003
T_1 / T_3	0.403	0.012	0.0001	0.001	0.0001	0.002
T_1 / T_4	0.028	0.147	0.001	0.082	0.0001	0.0001
T_2 / T_3	0.454	0.477	0.006	0.626	0.0001	0.0001
T_2 / T_4	0.361	0.854	0.130	0.282	0.0001	0.0001
T_3 / T_4	0.775	0.413	0.271	0.074	0.0001	0.0001

CPAP, continuous positive airway pressure; MAP, Mean arterial pressure.
